# N-glycomic profiling of colorectal cancer according to tumor stage and location

**DOI:** 10.1371/journal.pone.0234989

**Published:** 2020-06-29

**Authors:** Matilda Holm, Pirjo Nummela, Annamari Heiskanen, Tero Satomaa, Tuomas Kaprio, Harri Mustonen, Ari Ristimäki, Caj Haglund

**Affiliations:** 1 Department of Surgery, Faculty of Medicine, University of Helsinki and Helsinki University Hospital, Helsinki, Finland; 2 Department of Pathology, Faculty of Medicine, University of Helsinki and Helsinki University Hospital, Helsinki, Finland; 3 Translational Cancer Medicine Research Program, Faculty of Medicine, University of Helsinki, Helsinki, Finland; 4 Applied Tumor Genomics Research Program, Faculty of Medicine, University of Helsinki, Helsinki, Finland; 5 Glykos Finland Ltd., Helsinki, Finland; 6 HUSLAB, HUS Diagnostic Center, Helsinki University Hospital, Helsinki, Finland; Howard University, UNITED STATES

## Abstract

Alterations in glycosylation are seen in many types of cancer, including colorectal cancer (CRC). Glycans, the sugar moieties of glycoconjugates, are involved in many important functions relevant to cancer and can be of value as biomarkers. In this study, we have used mass spectrometry to analyze the N-glycan profiles of 35 CRC tissue samples and 10 healthy tissue samples from non-CRC patients who underwent operations for other reasons. The tumor samples were divided into groups depending on tumor location (right or left colon) and stage (II or III), while the healthy samples were divided into right or left colon. The levels of neutral and acidic N-glycan compositions and glycan classes were analyzed in a total of ten different groups. Surprisingly, there were no significant differences in glycan levels when all right- and left-sided CRC samples were compared, and few differences (such as in the abundance of the neutral N-glycan H3N5) were seen when the samples were divided according to both location and stage. Multiple significant differences were found in the levels of glycans and glycan classes when stage II and III samples were compared, and these glycans could be of value as candidates for new markers of cancer progression. In order to validate our findings, we analyzed healthy tissue samples from the right and left colon and found no significant differences in the levels of any of the glycans analyzed, confirming that our findings when comparing CRC samples from the right and left colon are not due to normal variations in the levels of glycans between the healthy right and left colon. Additionally, the levels of the acidic glycans H4N3F1P1, H5N4F1P1, and S1H5N4F1 were found to change in a cancer-specific but colon location-nonspecific manner, indicating that CRC affects glycan levels in similar ways regardless of tumor location.

## Introduction

Colorectal cancer (CRC) is the third most common cancer worldwide and has increased to become the second most common cause of death due to cancer, with over 1.8 million new cases and almost 900 000 deaths estimated to occur in 2018 [1]. While incidence and mortality rates are declining in certain countries, something thought to be at least partially due to early screening and prevention, the incidence of CRC is still predicted to increase by up to 60% by 2030 [1, 2]. 5-year survival rates for CRC can be as high as 60% in countries such as the United States, but are as low as 28% in developing countries [3].

The expression of glycans changes during cancer, and glycans are therefore of value as biomarkers and targets for new treatments [4]. Glycans are covalent assemblies of sugars that can exist either in free form as signaling molecules or as part of glycoconjugates such as glycoproteins. Most glycans exist as membrane-bound glycoconjugates [5]. They are found on all eukaryotic cell surfaces and are involved in many physiologically important functions such as cell signaling and adhesion, differentiation, and growth. Their complexity allows glycans on cell surfaces to function as signaling, recognition, and adhesion molecules [6, 7]. Malignant transformation is associated with changes in glycosylation, which include under- and overexpression of certain glycans, as well as neo-expression of glycans normally only expressed in embryonic tissues. The changes in glycosylation most often seen that are associated with cancer include sialylation, fucosylation, and N-linked glycan branching [4, 8]. Several previous studies have compared the serum/plasma asparagine-linked glycome (N-glycome) between CRC patients and healthy controls [9, 10]. The N-glycome of CRC tissues in comparison to healthy colon tissue has also been investigated in several studies. A study by Balog et al. found that structures with a bisecting N-acetylglucosamine were decreased in CRC, while sulfated glycans, paucimannosidic glycans, and sialylated Lewis type epitope-containing glycans were increased. This study also detected core-fucosylated mannose N-glycans in CRC samples [11]. We have also previously shown that sialylated N-glycans, paucimannose glycans, and small high-mannose type glycans are more common in rectal carcinomas than adenomas [12].

Multiple studies have found that right-sided colon cancer is associated with a worse prognosis than left-sided colon cancer [13, 14]. Differences between cancer in the right and left colon are also seen at the molecular level, with CRC in the right and left colon appearing to progress through different molecular pathways, with mutations in genes such as *KRAS* and *BRAF* being more common in right-sided tumors [15, 16]. Additionally, right- and left-sided CRC also have distinct mutational profiles [17]. Studies have also found significant differences in protein expression and plasma metabolites between patients with tumors in the right or left colon [18–23].

In this study, we have used matrix-assisted laser desorption/ionization time-of-flight (MALDI-TOF) mass spectrometry to analyze the N-glycan profiles of 35 tumor tissue samples from patients with CRC and 10 healthy colon tissue samples from patients who underwent operation for other reasons. The tumor tissue samples were divided into groups according to tumor location in the colon (right or left) and stage (II or III), while the healthy tissue samples were divided into two groups, right and left colon. This study provides new insight into how the levels of specific glycans and glycan classes differ depending on stage or location in the colon, as well as between healthy tissue and tumor tissue samples.

## Material and methods

### Study design

In this study, the N-glycan profiles of formalin-fixed, paraffin-embedded (FFPE) tumor tissue samples from 35 CRC patients and healthy colon tissue samples from 10 non-CRC patients were analyzed using a previously developed workflow [12, 24–27]. CRC patients were chosen according to the location and stage of their tumor. 18 patients had a tumor in the right colon and 17 in the left colon, with the division being made at the splenic flexure. Besides comparing right- and left-sided CRC, the glycan profiles were also compared between patients with stage II and III tumors.

### Tissue samples

Tissue samples from 35 CRC patients were selected for glycomic profiling. Patients with deaths due to reasons other than CRC were deliberately excluded from this study, as were patients with a previous history of non-colorectal cancer, hereditary nonpolyposis colorectal cancer, familial adenomatous polyposis, ulcerative colitis, Crohn’s disease, or celiac disease. In addition, 10 samples of healthy colon tissue from non-CRC patients were also selected. Five samples were from the right colon (caecum) and five from the left colon (sigma). These patients had undergone operations due to different issues affecting the colon (see S1 Table for details). The H&E slides of the tissue samples were confirmed by an experienced pathologist to be either unaffected or lightly affected by the cause of operation, and to display either completely or mostly normal histology. Patients with CRC were deliberately excluded when the healthy tissue samples were chosen. Detailed patient characteristics can be found in S1 Table. All tissue samples were routinely fixed and embedded in paraffin at the Department of Pathology, HUSLAB, Helsinki University Hospital, between 1996 and 2017. The Digital and Population Data Services Agency provided the follow-up data, and Statistics Finland provided the cause of death for all those deceased. This study was approved by the Surgical Ethics Committee of Helsinki University Hospital (Dnro HUS 226/E6/06, extension TMK02 §66 17.4.2013) and was carried out in accordance with the relevant guidelines and regulations. Written informed consent was obtained from all participants prior to sample collection.

### Glycan isolation

Glycans were isolated from FFPE tissue blocks as previously described [27]. To summarize, for the tumor tissue samples from CRC patients, representative areas of carcinoma tissue were marked on H&E slides and samples were punched from FFPE tissue blocks with a 3.0-mm puncher. For the healthy colon tissue samples, 10 μm flakes were cut using a microtome. The reason for using a microtome instead of a puncher for these samples was due to the better yield of epithelial cells made possible by sectioning. Whereas the FFPE blocks for the tumor tissue samples contained only tumor tissue, the samples from healthy colon tissue included not only epithelial cells but also other layers of the colon.

All samples were deparaffinized with xylene and an ethanol-water series according to standard procedures. N-linked glycans were detached from cellular glycoproteins by N-glycosidase F (PNGase F) digestion (Glyko; ProZyme Inc., Hayward, CA) and purified as previously described [12]. Briefly, the detached glycans were passed in water through C_18_ silica, after which they were absorbed to graphitized carbon material. Both of these steps were done in 96-well format. Next, the carbon wells were washed with water and neutral N-linked glycans were eluted using 25% acetonitrile, while acidic N-linked glycans were eluted using 0.05% trifluoroacetic acid in 25% acetonitrile in water. The acidic glycans were further purified through hydrophilic interaction solid-phase extraction done in 96-well format. Both glycan fractions were then passed in water through strong cation-exchange resin before MS analysis.

### Mass spectrometry

Matrix-assisted laser desorption/ionization time-of-flight (MALDI-TOF) mass spectrometry was performed using a Bruker Ultraflex III TOF/TOF mass spectrometer (Bruker Daltonics Inc, Bremen, Germany) as previously described [27]. Neutral N-glycans were detected in positive ion reflector mode as [M+Na]^+^ ions and acidic N-glycans in negative ion reflector mode as [M-H]^-^ ions. Two examples of unprocessed MALDI-TOF mass spectra of neutral and acidic N-glycans are given in S1 Fig (neutral N-glycans) and S2 Fig (acidic N-glycans). The relative molar abundances of neutral and acidic glycan components were assigned based on their relative signal intensities in the mass spectra when analyzed separately as neutral and acidic N-glycan fractions. The mass spectrometric raw data was transformed into the present glycan profiles as previously described [24, 25]. The resulting glycan signals in the glycan profiles were normalized to 100% to allow comparison between the samples and were assigned to biosynthetic groups based on their proposed monosaccharide composition, also as previously described [24, 25]. The mass spectrometry proteomics data have been deposited to the ProteomeXchange Consortium via the PRIDE [28, 29] partner repository with the dataset identifier PXD018673.

### Analysis of N-glycan profiles

For the tumor tissue samples, N-glycan data was analyzed in different groups according to the location of the tumor and tumor stage. Additionally, healthy tissue samples from the right and left colon were analyzed and the glycan profiles were compared both separately and together with the tumor tissue samples. Glycan abundance was analyzed separately within ten different groups, as seen in Table 1. Both specific monosaccharide compositions, and structural glycan classes were analyzed in these groups. The neutral and acidic N-glycan profiles were stratified by biosynthetic classification rules based on the amounts of hexose (H), N-acetylhexosamine (N), deoxyhexose (F), sialic acid (S), and sulfate/phosphate ester (P) residues in the proposed monosaccharide compositions. The classes were then shown as the proportion of major glycan structural classes between the different subgroups of samples.

**Table 1:** The ten groups within which N-glycan abundances were analyzed in this study.

Number	Group	Size
1	Right-sided CRC vs. left-sided CRC	18 vs. 17
2	Right-sided CRC vs. left-sided CRC, stage II samples only	9 vs. 9
3	Right-sided CRC vs. left-sided CRC, stage III samples only	9 vs. 8
4	Stage II vs. III CRC, all samples	18 vs. 17
5	Stage II vs. III CRC, right-sided samples only	9 vs. 9
6	Stage II vs. III CRC, left-sided samples only	9 vs. 8
7	Healthy colon tissue, right vs. left	5 vs. 5
8	Right colon, healthy tissue vs. CRC	5 vs. 18
9	Left colon, healthy tissue vs. CRC	5 vs. 17
10	Healthy colon tissue vs. CRC, all samples	10 vs. 35

For each group, relative N-glycan abundance was analyzed separately for both neutral and acidic N-glycans. N-glycan abundance was analyzed for the proposed monosaccharide compositions and, separately, for the proposed structural classes of glycans.

### Statistical analysis

For statistical analyses, the Mann-Whitney U test was used to analyze the values of the relative intensities of the N-glycan signals in order to compare the differences between the groups. P-values of < 0.05 were considered to be statistically significant. The false discovery rate (FDR) was controlled using the Benjamini-Hochberg method [30]. The Mann-Whitney U test and FDR correction were performed using SPSS version 24.0 (IBM SPSS Statistics, IBM Corporation, Armonk, NY). Orthogonal Projections to Latent Structures Discriminant Analysis (OPLS-DA) modeling was performed using the ropls [31] R package. For OPLS-DA modeling, both neutral and acidic proposed monosaccharide compositions with non-FDR corrected p-values of less than 0.05 were used in the same model.

## Results

### Glycomic profiling of colon cancer tissue samples

#### Neutral N-linked glycan profiles

**Fig 1:**
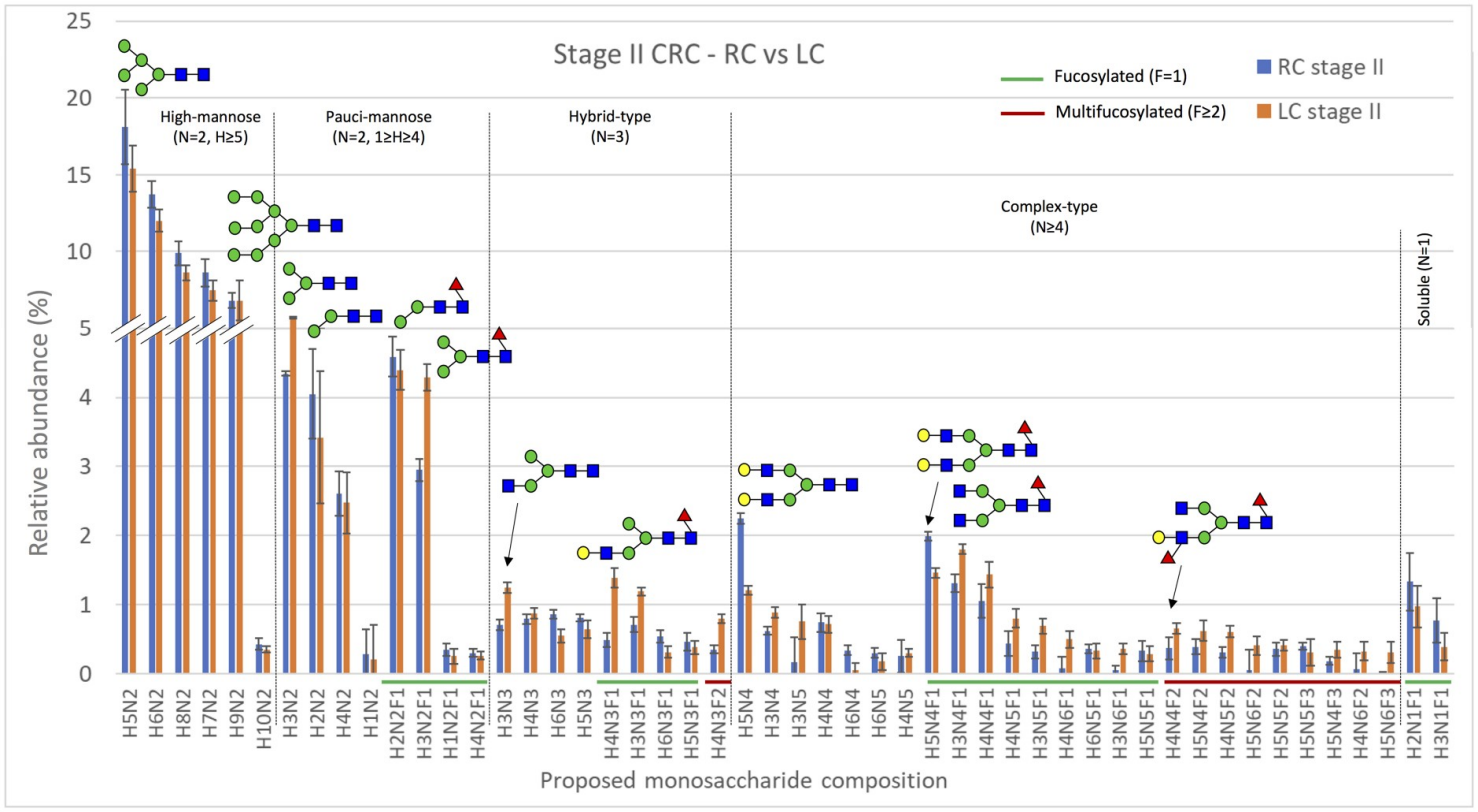
The relative abundance of the 50 most abundant neutral monosaccharide compositions in samples from patients with stage II cancer in the right (RC, blue bars, n = 9) or left (LC, orange bars, n = 9) colon. The x-axis shows the proposed neutral N-glycan monosaccharide composition, where fucosylated and multifucosylated N-glycans are highlighted with green or red lines, respectively. The results are shown as means ± SEM. Several putative N-glycan structures are depicted using symbols, with green circles representing D-mannose, blue squares representing N-acetyl-D-glucosamine, red triangles representing L-fucose, and yellow circles representing D-galactose. Major structural subgroups are separated by a dotted line. H = hexose, N = N-acetylhexosamine, F = fucose. Schematic glycan drawings are proposed based on known glycan structures detected in CRC tissues [11] and they were not validated by structural analyses in the present study.

The neutral N-linked glycan profiles of 35 CRC samples were analyzed using MALDI-TOF mass spectrometry. The levels of specific neutral N-glycan signals (assigned to proposed monosaccharide compositions) as well as the proposed structural classes of neutral glycan signals were compared separately within all seven groups of CRC patients studied, which are listed in Table 1. The neutral N-glycans expressed in the group comparing samples from patients with stage II cancer in the right or left colon are shown as the relative abundance of the 50 most abundant proposed monosaccharide compositions in Fig 1. The monosaccharide compositions H5N2, H6N2, H8N2, H7N2, and H9N2, which were classified as high-mannose N-glycans, were the most abundant neutral N-glycan compositions in all groups analyzed.

*Differences in glycan levels between right- and left-sided CRC*. When the levels of neutral monosaccharide compositions were analyzed according to tumor location (right or left colon), only the abundance of one glycan, with the proposed monosaccharide composition H3N5, was significantly different (p = 0.010) between the groups studied (all samples, stage II samples only, and stage III samples only). The relative abundance of H3N5 was significantly higher in left-sided stage II CRC when compared to right-sided stage II CRC (S2 Table). The neutral N-glycan profiles were also analyzed by grouping glycans with similar monosaccharide compositions into structural classes and calculating their combined proportions, similarly to as previously described [12, 25]. When the levels of these different classes of neutral glycans were compared between right- and left-sided CRC, no statistically significant differences were found (S3 Table). The abundances of the glycan classes were analyzed between all right- and left-sided CRC samples regardless of stage, as well as separately for stage II and stage III samples.

*Differences in glycan levels between stage II and III CRC*. The levels of neutral monosaccharide compositions were also compared between all stage II and III samples, in right-sided samples only, and in left-sided samples only in order to study changes in glycan levels during CRC progression. There were no statistically significant differences in the levels of either neutral N-glycans (S2 Table) or neutral N-glycan classes (S3 Table) when samples from patients with stage II and III CRC were compared.

#### Acidic N-linked glycan profiles

**Fig 2:**
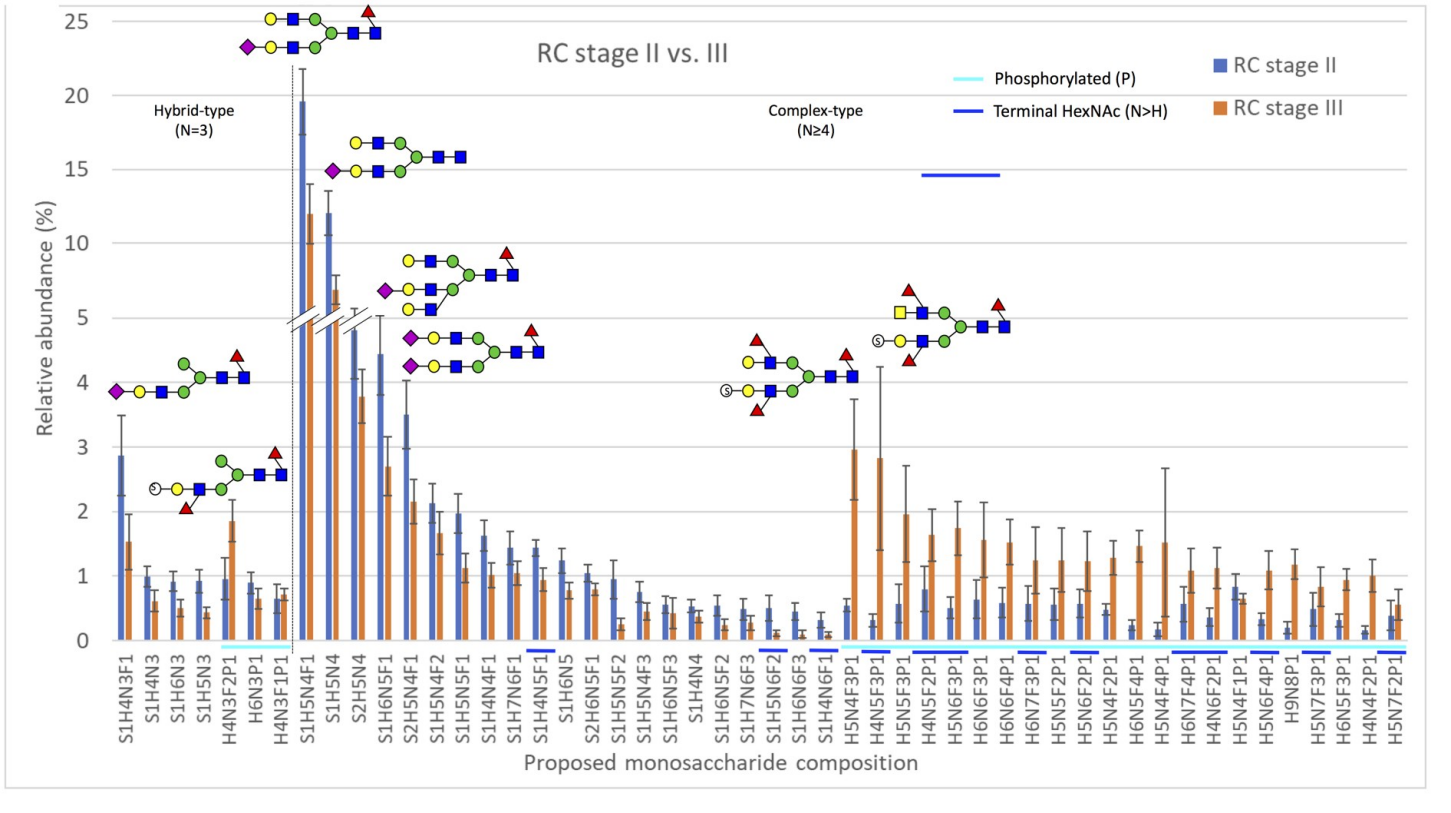
The relative abundance of the 50 most abundant acidic monosaccharide compositions in samples from patients with stage II (n = 9) or stage III (n = 9) cancer in the right colon. The x-axis shows the proposed acidic N-glycan monosaccharide composition, where sulfated/phosphorylated N-glycans are highlighted with a light blue line and N-glycans putatively containing a terminal N-acetylhexosamine (N>H) are highlighted with a dark blue line. The results are shown as means ± SEM. Several putative N-glycan structures are depicted using symbols, with green circles representing D-mannose, blue squares representing N-acetyl-D-glucosamine, red triangles representing L-fucose, yellow circles representing D-galactose, purple diamonds representing sialic acid (N-acetylneuraminic acid), and open circles marked with an “S” representing sulfate ester. Major structural subgroups are separated by a dotted line. H = hexose, N = N-acetylhexosamine, F = fucose, S = sialic acid, P = acid ester. Schematic glycan drawings are proposed based on known glycan structures detected in CRC tissues [11] and they were not validated by structural analyses in the present study.

**Fig 3:**
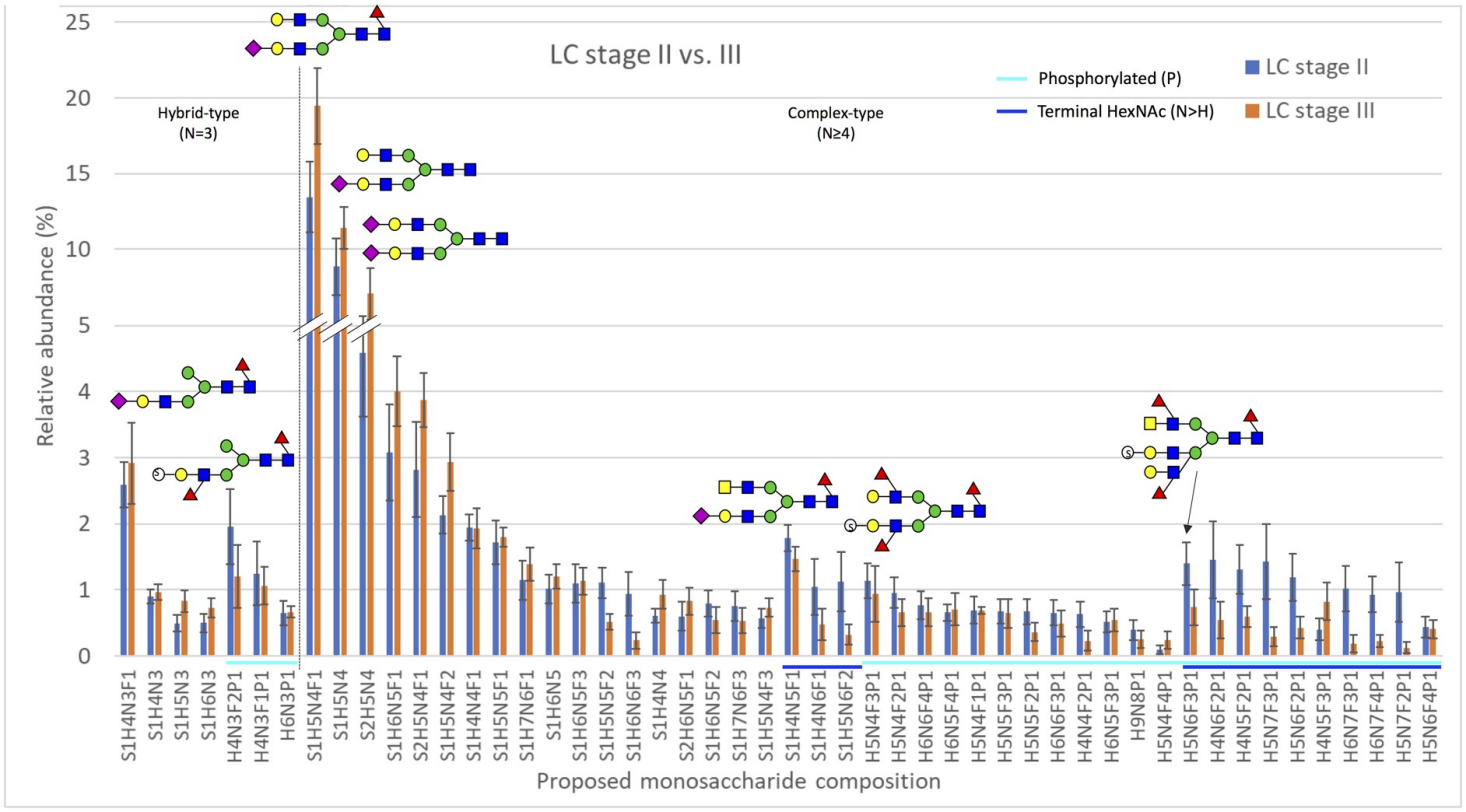
The relative abundance of the 50 most abundant acidic monosaccharide compositions in samples from patients with stage II (n = 9) or stage III (n = 8) cancer in the left colon. The x-axis shows the proposed acidic N-glycan monosaccharide composition, where sulfated/phosphorylated N-glycans are highlighted with a light blue line and N-glycans putatively containing a terminal N-acetylhexosamine (N>H) are highlighted with a dark blue line. The results are shown as means ± SEM. Several putative N-glycan structures are depicted using symbols, as specified in the legend of Fig 2. Major structural subgroups are separated by a dotted line. Schematic glycan drawings are proposed based on known glycan structures detected in CRC tissues [11] and they were not validated by structural analyses in the present study.

The profiles of acidic N-linked glycans, which contain acid esters (sulfate or phosphate) or sialic acid residues, were analyzed separately from the neutral N-glycans. The levels of specific acidic N-glycan signals as well as proposed structural classes of acidic glycans were compared separately within all seven groups of CRC patients studied (Table 1), as was done for neutral N-glycans. The levels of acidic N-glycans when compared between the groups stage II vs. III CRC, right-sided samples only (Fig 2) and stage II vs. III CRC, left-sided samples only (Fig 3) are shown as the relative abundance of the 50 most abundant proposed monosaccharide compositions. The monosaccharide compositions S1H5N4F1, S1H5N4, S2H5N4, S1H6N5F1, and S1H6N5F1, which were identified as sialylated complex-type N-glycans, were often found to be the most abundant glycan signals in the groups analyzed.

*Differences in glycan levels between right- and left-sided CRC*. When the levels of acidic monosaccharide compositions were analyzed according to tumor location, regardless of stage, no statistically significant differences between the levels of acidic N-glycans were found. The levels of acidic glycans were also analyzed between right- and left-sided CRC for stage II and III CRC samples separately, but no statistically significant differences were observed (S4 Table). The acidic N-glycan profiles were also analyzed by grouping glycans with similar monosaccharide compositions into structural classes and calculating their combined proportions, as previously described [12, 25] and as was done for the neutral N-glycans. When comparing the levels of the structural classes of acidic N-glycans between all right- and left-sided samples and, separately, stage II samples only, no statistically significant differences were seen (S5 Table). However, several statistically significant differences were seen between the levels of three classes of acidic glycans when compared between right- and left-sided stage III CRC. The levels of overall sialylation and sialylated complex-type N-glycans were significantly higher (Table 2) in left-sided than right-sided stage III CRC (S5 Table). On the other hand, the relative proportions of N-glycans with a sulfate/phosphate residue were significantly lower in left-sided stage III CRC when compared to right-sided stage III CRC (S5 Table). OPLS-DA modeling showed a clear separation between samples from patients with stage III cancer in the right or left colon (S3 Fig).

**Table 2:** Significantly different N-glycan classes (p < 0.05) when tumor tissue samples from CRC patients were compared.

Glycan class	Group	Neutral or acidic	Fold change	FDR-adjusted p-value
Overall sialylation	**Stage II** vs III CRC, right-sided samples only	Acidic	1,65	0,044
Overall sialylation	Right-sided CRC vs. **left-sided CRC**, stage III samples only	Acidic	1,67	0,037
Sialylated complex N-glycans	**Stage II** vs III CRC, right-sided samples only	Acidic	1,66	0,045
Sialylated complex N-glycans	Right-sided CRC vs. **left-sided CRC**, stage III samples only	Acidic	1,67	0,037
Sulfate/phosphate	Stage II vs **III** CRC, right-sided samples only	Acidic	2,40	0,044
Sulfate/phosphate	**Right-sided CRC** vs. left-sided CRC, stage III samples only	Acidic	2,43	0,037

Additional details can be found in S4 Table. The subgroup within which the levels of a glycan class are higher are highlighted in bold.

*Differences in glycan levels between stage II and III CRC*. When the levels of acidic monosaccharide compositions were compared between all stage II and III samples, significant differences in the levels of four glycans were seen (Table 3). The relative abundance of the sulfate/phosphate-containing and multifucosylated structure H4N5F3P1 was higher in stage III CRC, while the relative levels of the sialylated and multifucosylated structures S1H6N6F2, S1H6N7F3, and S1H7N7F4 were lower in stage III CRC when compared to stage II CRC (S4 Table). Interestingly, while the relative abundance of S1H7N7F4 was 0,25 in samples from stage II CRC patients, this composition was not detected at all in any samples from stage III patients. When focusing on samples from tumors in the right colon, the abundance of four monosaccharide compositions was significantly different between stage II and III CRC (Table 3).

**Table 3:** Significantly different proposed monosaccharide compositions (p < 0.05) when tumor tissue samples from CRC patients were compared.

Composition	Group	Neutral or acidic	Fold change	FDR-adjusted p-value
H3N5	Right-sided CRC vs. **left-sided CRC**, stage II samples only	Neutral	4,66	0,01
H5N4F3P1	Right-sided CRC, stage II vs. **III**	Acidic	5,43	0,009
H4N5F3P1	Stage II vs **III** CRC, all samples	Acidic	5,27	0,043
H4N5F3P1	Right-sided CRC, stage II vs. **III**	Acidic	9,04	0,022
H6N5F4P1	Right-sided CRC, stage II vs. **III**	Acidic	6,16	0,009
S1H6N6F2	**Stage II** vs III CRC, all samples	Acidic	4,31	0,044
S1H6N7F3	**Stage II** vs III CRC, all samples	Acidic	15,18	0,038
H9N8P1	Right-sided CRC, stage II vs. **III**	Acidic	6,02	0,019
S1H7N7F4	**Stage II** vs III CRC, all samples	Acidic	N/A	0,038

Additional details can be found in S1 and S3 Tables. The subgroup within which the levels of a specific monosaccharide composition are higher are highlighted in bold. The fold change for the composition S1H7N7F4 is not available, as the relative abundance was 0.25 in stage II samples, while S1H7N7F4 was not detected at all in any of the stage III samples (with a relative abundance of 0.00).

The sulfate/phosphate-containing glycans H5N4F3P1, H4N5F3P1, H6N5F4P1, and H9N8P1 all displayed higher relative levels in stage III CRC, indicating that the relative abundance of these glycans changes when CRC progresses from local to regionally advanced disease (S4 Table). In samples from the left colon, no statistically significant differences in the relative levels of any acidic glycans were found between stage II and III CRC (S4 Table). Overall, more differences were observed between the groups analyzed for acidic N-glycans than neutral N-glycans. The levels of multiple monosaccharide compositions were significantly different within two groups, namely stage II vs. III, all samples, and stage II vs. III, right-sided samples only (S4 Table). The overexpression of the sulfate/phosphate-containing and multifucosylated structure H4N5F3P1 was significant in both of these groups.

When comparing the classes of acidic N-glycans between all stage II and III samples regardless of tumor location, no statistically significant differences in the levels of acidic glycans were seen (S5 Table). When the classes of acidic N-glycans were compared between stage II and III right-sided CRC, significant differences between the relative proportions of the same three glycan classes whose levels differed between right- and left-sided stage III CRC (overall sialylation, sialylated complex N-glycans, and N-glycans with a sulfate/phosphate residue) were seen (Table 2). The relative levels of overall sialylation and sialylated complex N-glycans were significantly decreased in stage III right-sided CRC (S5 Table). On the other hand, the relative levels of N-glycans with a sulfate/phosphate residue were significantly increased in stage III right-sided CRC (S5 Table). When the relative levels of acidic glycan classes were compared between stage II and III samples from the left colon, no statistically significant differences were found (S5 Table). These results indicate that at least in right-sided CRC, cancer progression from stage II to III is associated with significant changes in the abundance of certain classes of acidic glycans.

### Glycomic profiling of healthy colon tissue samples

#### Neutral N-linked glycan profiles

Healthy colon tissue samples from 10 patients were also analyzed and the levels of neutral N-glycan compositions and glycan classes were compared between samples from the right colon (n = 5) and left colon (n = 5). None of the differences in the levels of glycans and glycan classes were statistically significant after FDR correction (S2 and S3 Tables).

#### Acidic N-linked glycan profiles

When the levels of acidic N-glycan compositions and N-glycan classes were analyzed in healthy tissue samples from the right and left colon, no statistically significant differences were observed (S4 and S5 Tables).

### Comparison of glycan profiles between healthy and tumor tissue

#### Neutral N-linked glycan profiles

When healthy tissue samples from the right colon were compared with tumor tissue samples from the right colon, no significant differences were seen in the levels of any neutral N-glycan compositions (S2 Table). However, the levels of fucosylation were significantly higher in tumor tissue samples from the right colon when compared to healthy colon tissue (S3 Table). When healthy tissue samples from the left colon were compared with tumor tissue samples from the left colon, the levels of 20 out of 126 neutral N-glycan compositions were significantly different between the two groups (S2 Table), and the levels of paucimannose type N-glycans were significantly higher in tumor tissue samples (S3 Table). All healthy colon tissue samples were also compared to all CRC tissue samples. The levels of 21 neutral N-glycan compositions were significantly different between the groups (S2 Table). Additionally, the levels of both paucimannose type N-glycans and fucosylation were significantly higher in CRC samples than healthy colon tissue samples (S3 Table).

#### Acidic N-linked glycan profiles

When healthy tissue samples from the right colon were compared with tumor tissue samples from the right colon, differences in the levels of five out of 113 acidic N-glycan compositions were significant (S4 Table). Healthy tissue samples from the left colon were also compared to tumor tissue samples from the left colon and significant differences in the levels of 12 acidic N-glycan compositions were found (S4 Table). When all healthy tissue samples were compared to all CRC samples, the levels of 74 of 113 acidic N-glycan compositions had significantly different levels between the two groups (S4 Table). Significant differences were seen in the same 11 out of 14 N-glycan classes when their levels were compared between healthy colon tissue and tumor tissue samples in only samples from the right colon, only samples from the left colon, and in all samples combined (S5 Table). Differences include levels of complex fucosylation and sulfate/phosphate-containing N-glycans being significantly higher in CRC when compared to healthy colon tissue, with levels of biantennary-size N-glycans and sialylation being significantly lower in tumor tissue samples.

## Discussion

In this study, we used MALDI-TOF MS to analyze the N-linked glycan profiles of 35 CRC tissue samples from patients with right- and left-sided CRC that were also divided into groups based on tumor stage (II or III). Due to the large number of monosaccharide compositions detected, we used false discovery rate (FDR) correction to control false positive results in statistical analyses. Surprisingly, there were no significant differences between the levels of neutral and acidic N-glycans or structural N-glycan classes expressed when all right- and left-sided CRC samples were compared. Only one neutral N-glycan, H3N5, displayed significantly different levels (p = 0.010) between right- and left-sided stage II CRC. The levels of overall sialylation, sialylated complex acidic N-glycans, and sulfated/phosphorylated glycans differed between right- and left-sided stage III CRC, and these subgroups could be clearly separated using OPLS-DA modeling (S3 Fig). Interestingly, these differences were only seen when samples were stratified according to stage, with no significant differences in glycan levels seen between overall right- and left-sided CRC.

We found significant differences in the relative levels of four acidic N-glycans (H4N5F3P1, S1H6N6F2, S1H6N7F3, and S1H7N7F4) between all stage II and III samples and in the relative levels of four acidic N-glycans (H5N4F3P1, H4N5F3P1, H6N5F4P1, and H9N8P1) between stage II and III samples from the right colon (S4 Table). H4N5F3P1 displayed significantly higher levels in stage III CRC in both of the above groups, indicating that it may be linked to CRC progression and regional metastasis. In left-sided tumors, the abundance of H4N5F3P1 also increased from stage II to III, but the increase was not significant. H4N5F3P1 therefore has the potential to function as a target for new therapies after further investigation into its role in CRC progression. The proposed monosaccharide composition of this glycan signal comprises several interesting features, namely sulfation/phosphorylation (P), multifucosylation (F>1), and potential terminal N-acetylhexosamine (N>H), all of which have been previously linked to CRC [11, 12, 25]. Structural analysis of this glycan is thus warranted for example in order to identify potential epitopes for anti-glycan antibody targeting.

Three structural classes of acidic N-glycans, overall sialylation, sialylated complex glycans, and sulfated/phosphorylated glycans, also displayed significantly altered levels between stage II and III CRC in the right colon (Table 2), indicating that these changes may be linked to cancer progression. Further studies are needed to elucidate if these glycans play a role in regional metastasis and if patients could benefit from new therapies targeting these glycans. Interestingly, there were no significant changes in the levels of either neutral (S3 Table) or acidic (S5 Table) glycan classes when all stage II and III samples were compared. These findings indicate that there are no widespread changes in classes of glycans during overall CRC progression, despite the changes seen as right-sided CRC progressed from stage II to III.

In order to validate our findings that the levels of H3N5 and three acidic glycan classes differed between right- and left-sided CRC (in stage II and stage III samples, respectively), we also analyzed healthy tissue samples from the right and left colon. There were no significant differences in any of the neutral (S2 and S3 Tables) or acidic (S4 and S5 Tables) glycans and glycan classes analyzed when these samples were compared. It is thereby possible to confirm that our findings when comparing tumor tissue samples from the right and left colon are not due to normal variations in the levels of glycans between the healthy right and left colon. Additionally, the levels of acidic glycans such as H4N3F1P1, H5N4F1P1, and S1H5N4F1 were significantly different when healthy tissue samples from the right or left colon were compared with tumor tissue samples from the corresponding location, as well as when all healthy tissue samples were compared to all tumor tissue samples. The levels of these three glycans did not differ significantly when compared between healthy tissue samples from the right and left colon, or when compared between CRC tissue samples from the right and left colon. These results indicate that the changes in the levels of H4N3F1P1, H5N4F1P1, and S1H5N4F1 are cancer-specific, although not colon location-specific.

Our findings suggest that while the normal right and left colon differ physiologically [16], these differences do not appear to the same extent at glycan level, as there were no significant differences in the relative levels of glycans or glycan classes between mass spectrometric N-glycan profiles when healthy tissue samples from the right or left colon were compared (S2–S5 Tables). It appears that CRC affects N-glycan profiles in similar ways regardless of where in the colon the tumor arises, as we found that the relative proportions of the same 11 acidic glycan classes were significantly different when healthy tissue samples were compared to tumor tissue samples both separately according to location in the colon (right or left) and when all healthy samples were compared to all cancer samples (S5 Table). Additionally, no significant differences were seen when N-glycan profiles were compared between all right- and left-sided CRC samples, which lends further support to this hypothesis. It is known that cancer directly affects glycan expression, which can also be seen in the large overall changes in relative N-glycan levels between healthy colon tissue and tumor tissue observed here, especially among acidic glycans and glycan classes (S4 and S5 Tables), but also among neutral glycan compositions (S2 Table) and, to a lesser extent, neutral glycan classes (S3 Table). These N-glycans and N-glycan classes, whose relative levels significantly differ between healthy colon tissue and CRC tissue, could be of value as new diagnostic markers and new targets for anticancer therapies after further validation.

We have previously shown that N-glycomic profiling can be used to separate rectal adenomas from carcinomas [12]. In that study, differences were seen in glycosylation between stage I-II and stage III carcinomas, as well as between stage I-II and stage III-IV carcinomas. The inclusion of adenomas and focus on comparing adenomas and carcinomas in our previous study made comparisons to our current study difficult. However, some similarities were seen between the two studies. The high-mannose type glycans H5N2, H6N2, H7N2, H8N2, and H9N2, which were the most abundant neutral glycans in both rectal adenomas and carcinomas, were also the most abundant in carcinoma samples from the right and left colon. In our previous study, acidic N-glycans with five N-acetylhexosamine residues (compositions containing N5, such as H4N5F1P1 and H4N5F2P1) were more abundant in stage III than stage I-II rectal carcinomas. In this study, while no significant differences were found between stage II and III CRC samples from the left colon, changes in the relative levels of two glycans containing N5, namely H4N5F3P1 and H6N5F4P1, were seen when right-sided stage II and III samples were compared. This further indicates that acidic glycans containing N5 may play roles in the progression of CRC from local to regional disease.

Several previous studies have investigated altered glycosylation in CRC. A study by Sethi *et al*. reported an overrepresentation of high-mannose and paucimannose N-glycans in CRC as compared to adjacent normal colon tissue [32]. Increased levels of mannose-type N-glycans have been suggested to be a key molecular feature of CRC [33]. High-mannose type glycans were also the most abundant neutral N-glycans in our set of 35 CRC tissue samples. Increased sialylation has previously been implicated in CRC metastasis, therapeutic resistance, and disease recurrence [34–36]. In our study, sialylation was the most abundant acidic N-glycan modification in most of the samples, except in right-sided stage III tumors, where sulfate/phosphate esters dominated. As mentioned earlier, a study by Balog et al. discovered that structures with a bisecting N-acetylglucosamine were decreased in CRC as compared to corresponding control tissue samples, while paucimannose glycans and glycans that could potentially contain sialylated Lewis-type epitopes (sialylated glycans with at least two fucose residues) were increased in CRC. In our current study, we found that the composition H5N5, whose proposed structure contains a bisecting N-acetylglucosamine, as well as H3N6F2 and H3N6F3, whose proposed structures contain a terminal N-acetylhexosamine, all had significantly lower relative levels in CRC when tumor tissue samples were compared to healthy tissue samples (S2 Table). Additionally, the relative levels of the paucimannosidic compositions H2N2F1 and H3N2F1 were significantly higher in tumor tissue samples when compared to healthy colon tissue samples (S2 Table). We also identified multiple compositions that could potentially contain sialylated Lewis-type structures, including S1H4N5F2, S1H5N4F3, S1H5N5F2, S1H6N5F2, S1H5N6F2, S1H7N6F3, and S1H7N6F4. The relative levels of all of these glycans were significantly higher in tumor tissue samples when compared to healthy tissue samples (S4 Table). These findings are all in concordance with the findings of Balog et al., further indicating the importance of these glycans in CRC.

Multiple previous studies of the CRC N-glycome have utilized cell lines or serum/plasma samples from patients [37–40]. This study was strengthened by the use of tumor tissue samples, which made it possible to directly study tumor-derived glycans and CRC progression. It also enabled the correlation of changes in glycan levels to clinical stage. Additional strengths of this study were the relatively large number of samples analyzed and the FDR correction used to control false positives in the statistical analyses. One limit of this study is that although the glycans were detached from tumor-derived glycoproteins, with the methods used, it is not possible to determine which specific cell types (e.g. cancer or stromal/immune cells) they originally came from. This study identified multiple glycans whose levels differed between stage II and III CRC, which could be of value as biomarkers to predict disease progression, although further validation is needed. For example, by staining tumor tissue samples from patients with stage II for glycans whose levels were increased in stage III CRC, the risk of disease progression could be predicted. These high-risk stage II patients could therefore benefit from more aggressive treatment for example through adjuvant therapy, which is currently controversial for stage II patients [41]. Additional studies are also needed to elucidate why these changes in glycan abundance occur, which proteins these glycans are attached to, and the effects of altered glycan expression in CRC. This study provides new insights into the biological differences at glycan level between healthy colon and tumor tissue, as well as between right- and left-sided colon cancer and local and regional disease.

## Supporting information

S1 Fig The unprocessed MALDI-TOF mass spectrum of neutral N-linked glycans isolated from a tumor tissue sample from a patient with stage II cancer in the right colon. (PDF)

S2 Fig The unprocessed MALDI-TOF mass spectrum of acidic N-linked glycans isolated from a tumor tissue sample from a patient with stage II cancer in the right colon (the same patient as in S1 Fig). (PDF)

S3 Fig OPLS-DA modeling showing the separation between samples from patients with stage III cancer in the right or left colon. For this model, both significantly different (p < 0.05, non-FDR corrected) neutral and acidic proposed monosaccharide compositions were used. The ellipses represent the 95% of the multivariate normal distributions for each class shown.
(PDF)

S1 Table Information about the 35 CRC patients and 10 heathy tissue donors included in this study. (XLSX)

S2 Table The relative abundance of the proposed neutral N-glycans detected in the CRC and healthy colon tissue samples analyzed in the groups in this study. The relative abundance of each proposed monosaccharide composition is shown as the average ± standard error of the mean (SEM). The p-value for each proposed monosaccharide composition compared within all ten groups is also given.
(XLSX)

S3 Table The relative abundance of the neutral N-glycan classes detected in the CRC and healthy colon tissue samples analyzed in the groups in this study. The relative abundance of each N-glycan class is shown as the average ± SEM. The p-value for each N-glycan class compared within all ten groups is also given.
(XLSX)

S4 Table The relative abundance of the proposed acidic N-glycans detected in the CRC and healthy colon tissue samples analyzed in the groups in this study. The relative abundance of each proposed monosaccharide composition is shown as the average ± SEM. The p-value for each proposed monosaccharide composition compared within all ten groups is also given.
(XLSX)

S5 Table The relative abundance of the acidic N-glycan classes detected in the CRC and healthy colon tissue samples analyzed in the groups in this study. The relative abundance of each N-glycan class is shown as the average ± SEM. The p-value for each N-glycan class compared within all ten groups is also given.
(XLSX)
