# Supplementary figures and images for: N-glycomic profiling of colorectal cancer according to tumor stage and location
Source: PLoS One. 2020 Jun 29;15(6):e0234989. doi: 10.1371/journal.pone.0234989 (PMC7323945; doi:10.1371/journal.pone.0234989)

Supplementary  
Figure 1

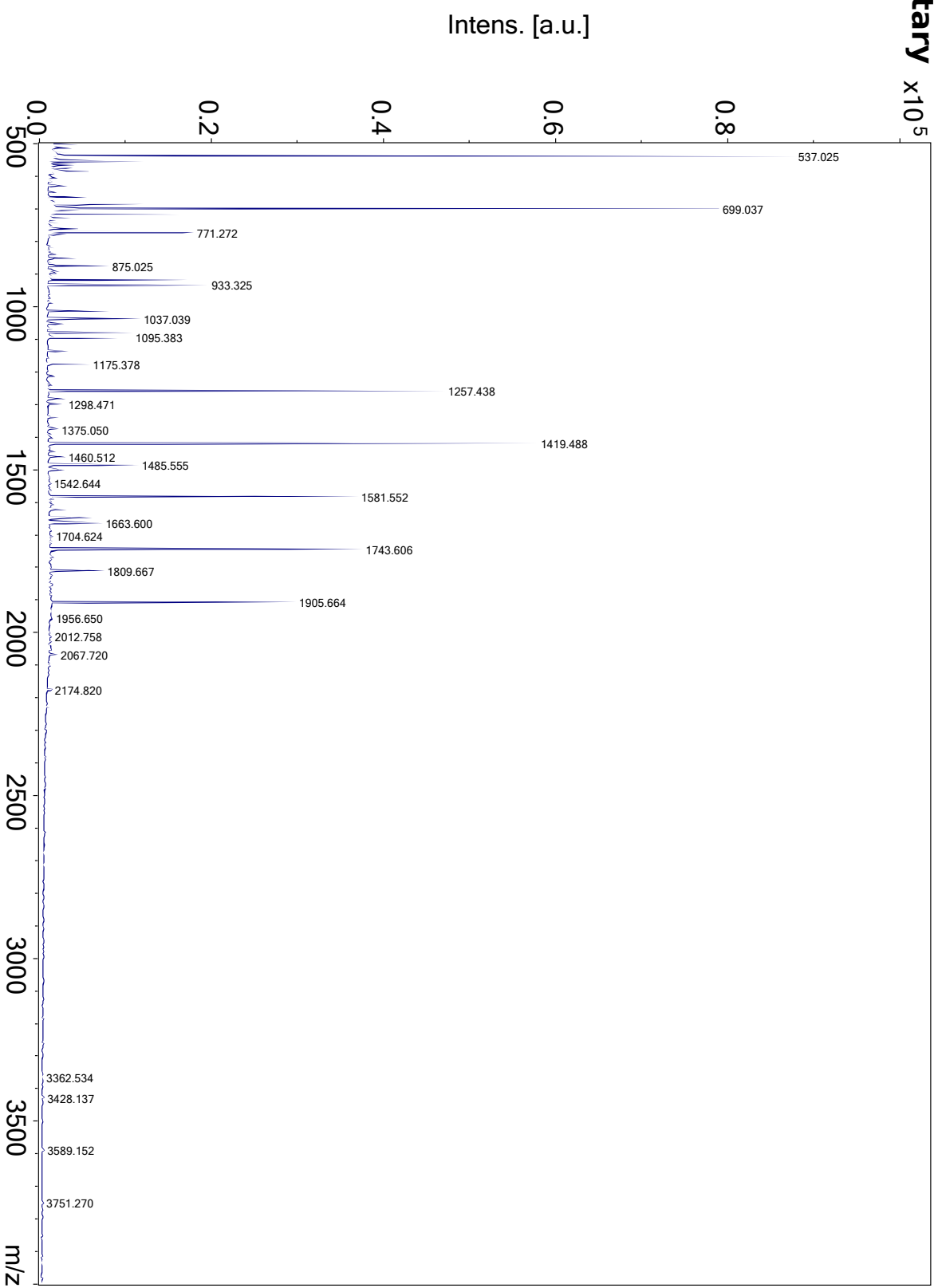

Sample 5  
(right colon  
stage II)

Supplement: S1 Fig — (PDF) [file pone.0234989.s001.pdf]

**Supplementary**  
**Figure 2**

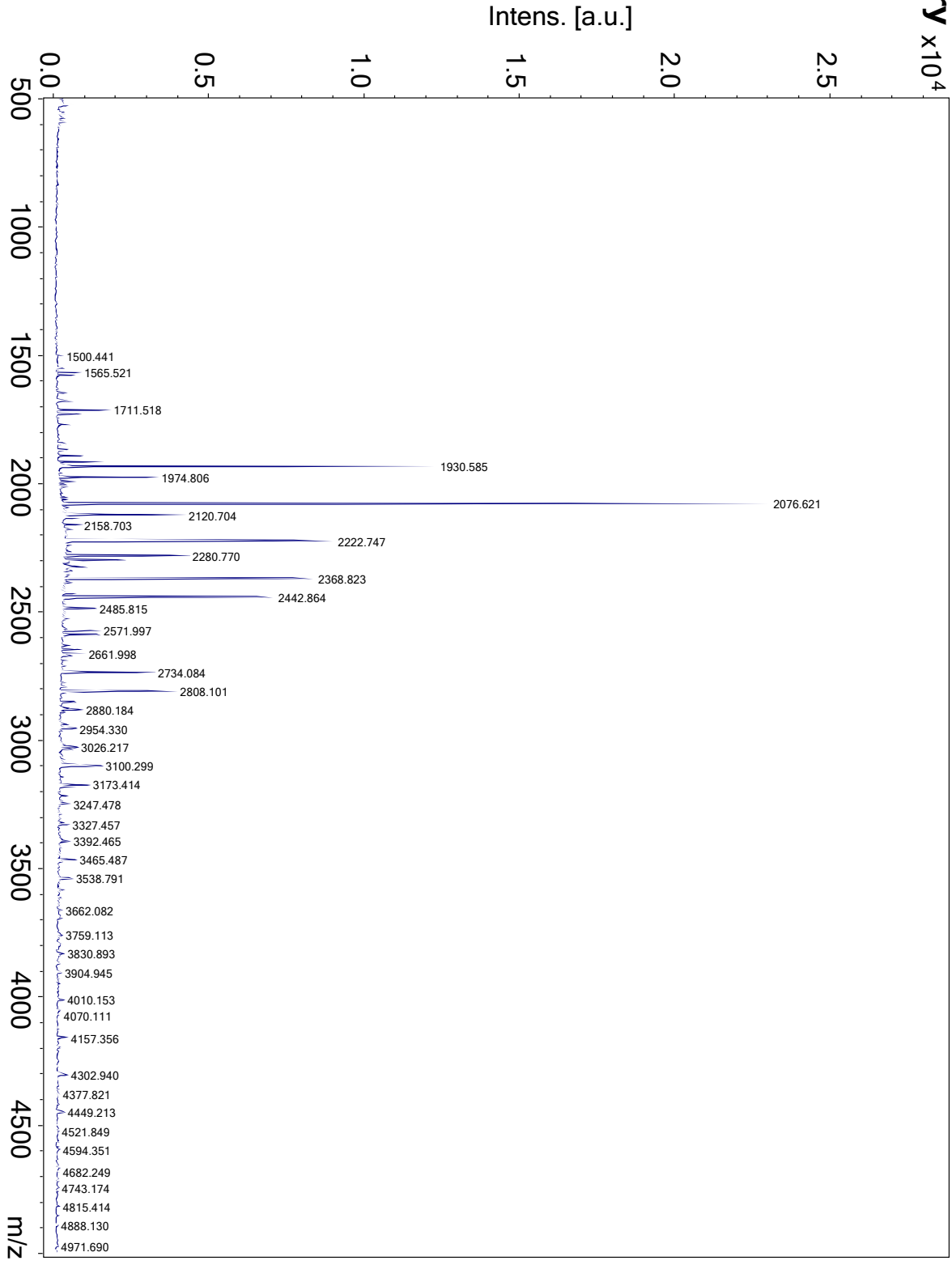

**Sample 5**  
**(right colon**  
**stage II)**

Supplement: S2 Fig — (PDF) [file pone.0234989.s002.pdf]

## Supplementary Figure 3

Scores (OPLS-DA)

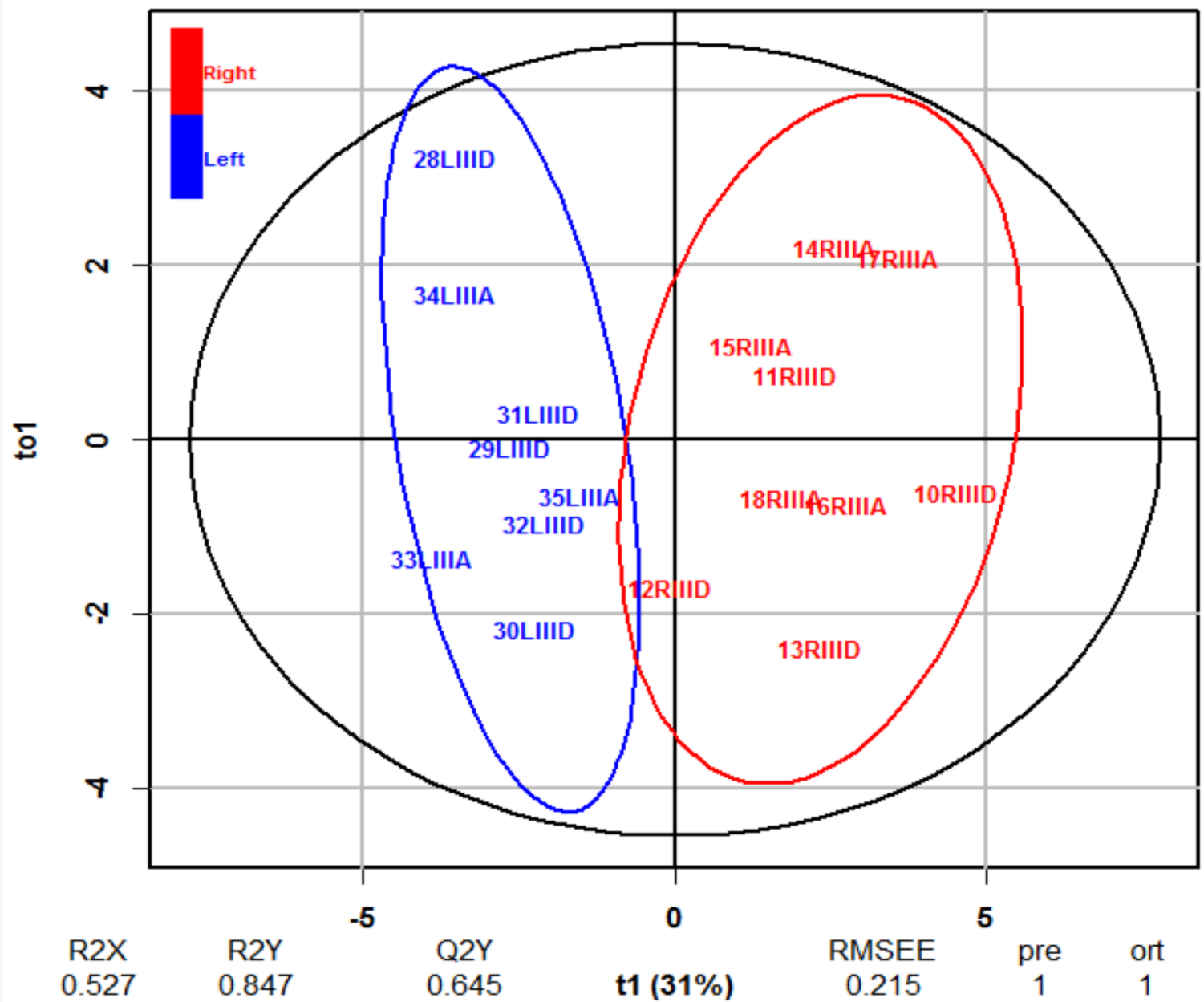

Supplement: S3 Fig — For this model, both significantly different (p < 0.05, non-FDR corrected) neutral and acidic proposed monosaccharide compositions were used. The ellipses represent the 95% of the multivariate normal distributions for each class shown. (PDF) [file pone.0234989.s003.pdf]
